# Supplementary material for: Arsenic disulfide promoted the demethylation of PTPL1 in diffuse large B cell lymphoma cells
Source: PeerJ. 2024 May 14;12:e17363. doi: 10.7717/peerj.17363 (PMC11100478; doi:10.7717/peerj.17363)
Supplement: Supplemental Information 1 [file peerj-12-17363-s001.docx]

**Supplementary File 1**

**1. PTPL1 mRNA expression**

|  | **Gene name** | **Mean** | **Gene name** | **Mean** |
| --- | --- | --- | --- | --- |
|  | *GAPDH* |  | *PTPL1* |  |
| Control | 17.14 | 17.479 | 18.08 | 17.722 |
| Control | 17.60 |  | 17.11 |  |
| Control | 17.69 |  | 17.98 |  |
| NC | 17.73 | 17.724 | 17.64 | 17.721 |
| NC | 17.62 |  | 18.02 |  |
| NC | 17.82 |  | 17.50 |  |
| SiRNA1 | 17.87 | 17.787 | 18.39 | 18.441 |
| SiRNA1 | 17.67 |  | 18.46 |  |
| SiRNA1 | 17.83 |  | 18.47 |  |
| SiRNA2 | 18.05 | 17.755 | 19.50 | 19.671 |
| SiRNA2 | 17.50 |  | 19.76 |  |
| SiRNA2 | 17.72 |  | 19.75 |  |
| SiRNA3 | 17.82 | 17.735 | 18.80 | 18.624 |
| SiRNA3 | 17.68 |  | 18.35 |  |
| SiRNA3 | 17.71 |  | 18.72 |  |

**2. Western blotting**

**Replicates for PTPL in DB cells:**


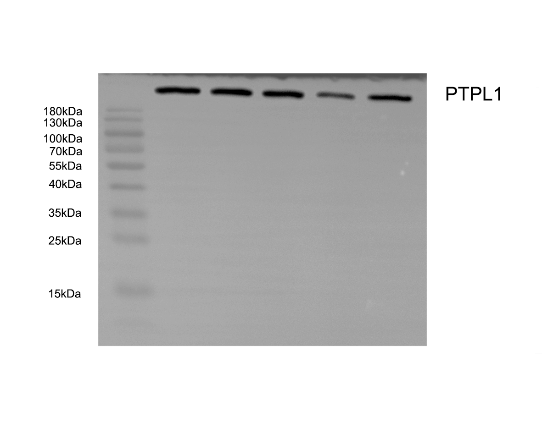

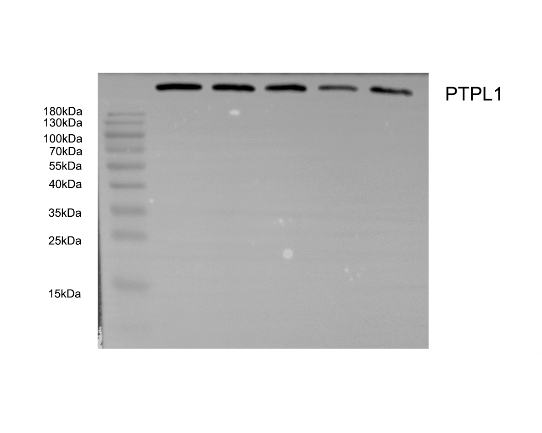


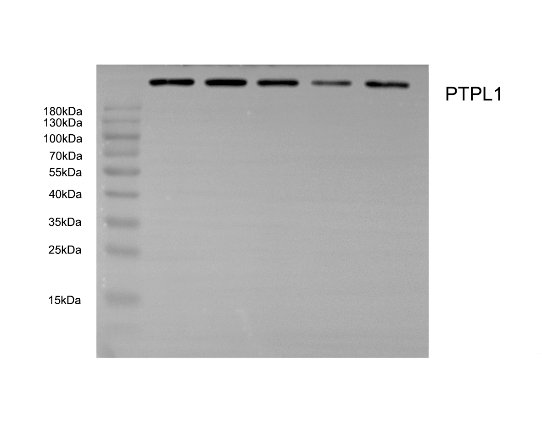


**Replicates for GAPDH in DB cells:**


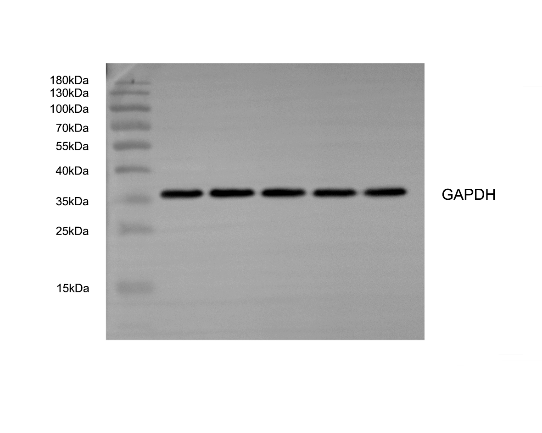

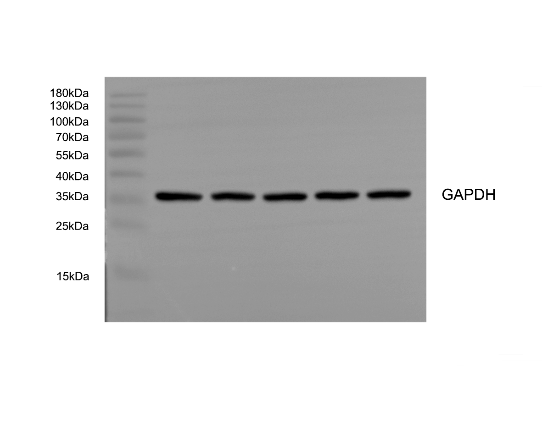


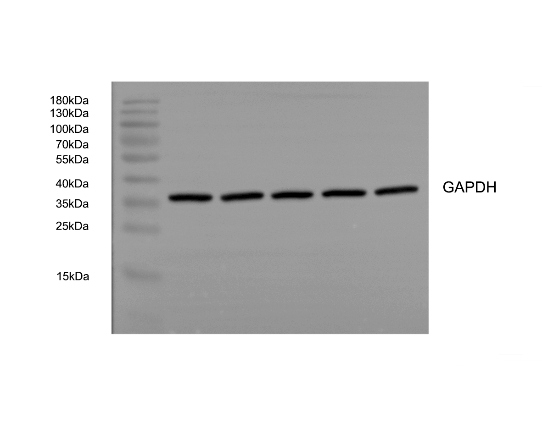


**Replicates for PTPL in SU-DHL-4 cells:**


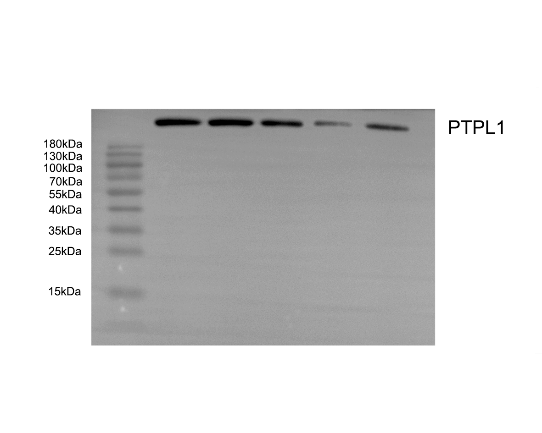

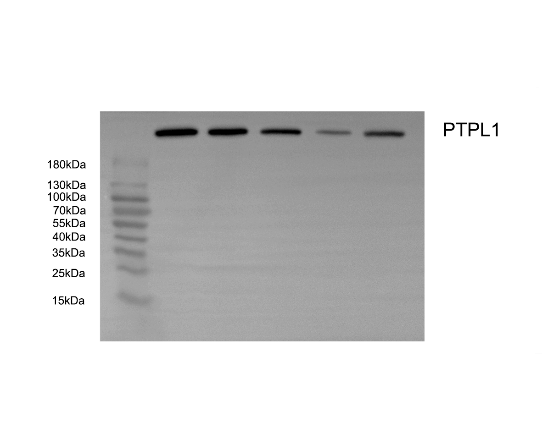


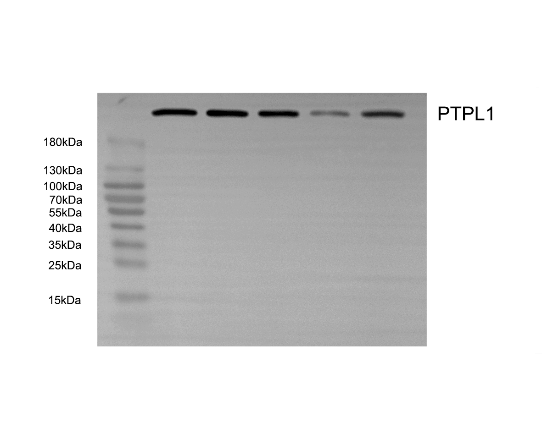


**Replicates for GAPDH in SU-DHL-4 cells:**


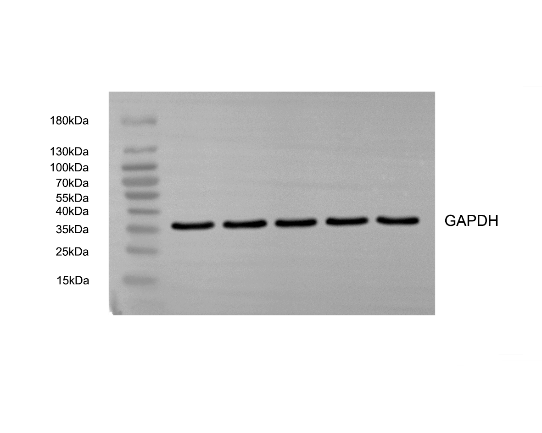

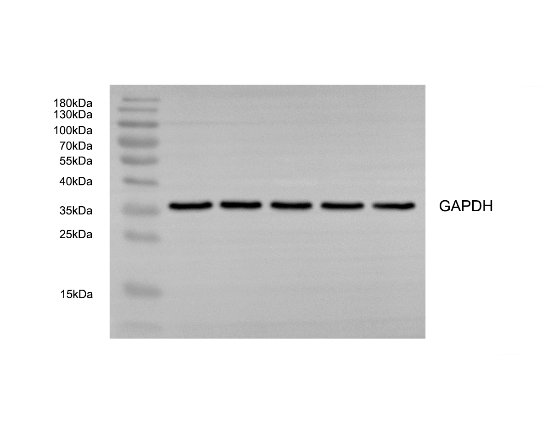


**3. PTPL1 protein expression in DB cells:**

| **Group** | **OD** | **OD-0** | **Sample concentration（mg/ml)** |
| --- | --- | --- | --- |
| Control（N=1） | 0.236 | 0.167 | 1.56 |
|  | 0.225 | 0.156 | 1.47 |
| NC（N=1） | 0.219 | 0.15 | 1.41 |
|  | 0.212 | 0.143 | 1.35 |
| SiRNA1（N=1） | 0.219 | 0.15 | 1.41 |
|  | 0.215 | 0.146 | 1.38 |
| SiRNA2（N=1） | 0.231 | 0.162 | 1.52 |
|  | 0.243 | 0.174 | 1.62 |
| SiRNA3（N=1） | 0.273 | 0.204 | 1.89 |
|  | 0.261 | 0.192 | 1.78 |
| Control（N=2） | 0.277 | 0.208 | 1.93 |
|  | 0.283 | 0.214 | 1.98 |
| NC（N=2） | 0.19 | 0.121 | 1.16 |
|  | 0.194 | 0.125 | 1.19 |
| SiRNA1（N=2） | 0.177 | 0.108 | 1.04 |
|  | 0.177 | 0.108 | 1.04 |
| SiRNA2（N=2） | 0.184 | 0.115 | 1.10 |
|  | 0.186 | 0.117 | 1.12 |
| SiRNA3（N=2） | 0.262 | 0.193 | 1.79 |
|  | 0.26 | 0.191 | 1.78 |
| Control（N=3） | 0.285 | 0.216 | 2.00 |
|  | 0.279 | 0.21 | 1.94 |
| NC（N=3） | 0.211 | 0.142 | 1.34 |
|  | 0.212 | 0.143 | 1.35 |
| SiRNA1（N=3） | 0.182 | 0.113 | 1.08 |
|  | 0.19 | 0.121 | 1.16 |
| SiRNA2（N=3） | 0.228 | 0.159 | 1.49 |
|  | 0.224 | 0.155 | 1.46 |
| SiRNA3（N=3） | 0.219 | 0.15 | 1.41 |
|  | 0.22 | 0.151 | 1.42 |

**4. PTPL1 protein expression in SU-DHL-4 cells:**

| **Group** | **OD** | **OD-0** | **Sample concentration（mg/ml)** |
| --- | --- | --- | --- |
| Control（N=1） | 0.254 | 0.185 | 1.72 |
|  | 0.254 | 0.185 | 1.72 |
| NC（N=1） | 0.263 | 0.194 | 1.80 |
|  | 0.259 | 0.19 | 1.77 |
| SiRNA1（N=1） | 0.278 | 0.209 | 1.93 |
|  | 0.287 | 0.218 | 2.01 |
| SiRNA2（N=1） | 0.287 | 0.218 | 2.01 |
|  | 0.266 | 0.197 | 1.83 |
| SiRNA3（N=1） | 0.232 | 0.163 | 1.53 |
|  | 0.247 | 0.178 | 1.66 |
| Control（N=2） | 0.279 | 0.21 | 1.94 |
|  | 0.292 | 0.223 | 2.06 |
| NC（N=2） | 0.187 | 0.118 | 1.13 |
|  | 0.175 | 0.106 | 1.02 |
| SiRNA1（N=2） | 0.198 | 0.129 | 1.23 |
|  | 0.2 | 0.131 | 1.24 |
| SiRNA2（N=2） | 0.131 | 0.062 | 0.63 |
|  | 0.133 | 0.064 | 0.65 |
| SiRNA3（N=2） | 0.154 | 0.085 | 0.84 |
|  | 0.155 | 0.086 | 0.85 |
| Control（N=3） | 0.233 | 0.164 | 1.54 |
|  | 0.223 | 0.154 | 1.45 |
| NC（N=3） | 0.249 | 0.18 | 1.68 |
|  | 0.228 | 0.159 | 1.49 |
| SiRNA1（N=3） | 0.276 | 0.207 | 1.92 |
|  | 0.277 | 0.208 | 1.93 |
| SiRNA2（N=3） | 0.212 | 0.143 | 1.35 |
|  | 0.217 | 0.148 | 1.39 |
| SiRNA3（N=3） | 0.257 | 0.188 | 1.75 |
|  | 0.273 | 0.204 | 1.89 |
