# Supplementary material for: Arsenic disulfide promoted the demethylation of PTPL1 in diffuse large B cell lymphoma cells
Source: PeerJ. 2024 May 14;12:e17363. doi: 10.7717/peerj.17363 (PMC11100478; doi:10.7717/peerj.17363)
Supplement: Supplemental Information 3 [file peerj-12-17363-s003.docx]

**Supplementary File 3**


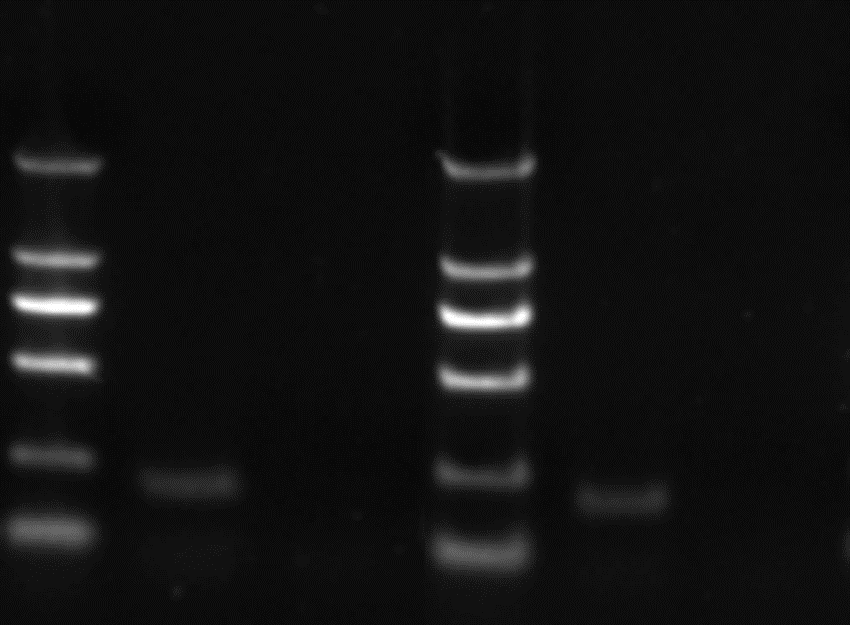


*PTPL1* methylation in DB and SU-DHL-4 cell lines detected by MSPCR.


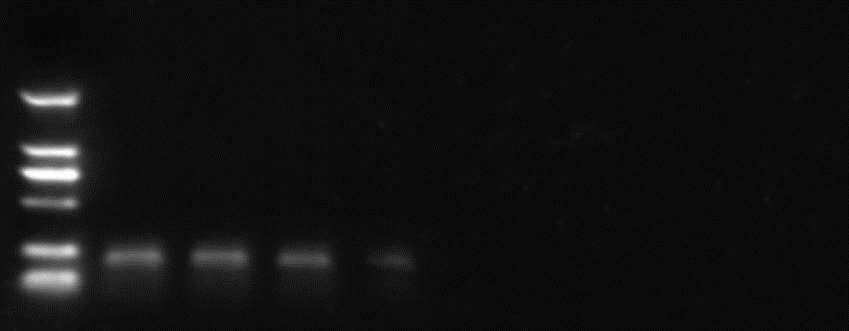


PTPL1 methylation in DB cells treated with different doses of arsenic disulfide detected by MSPCR.


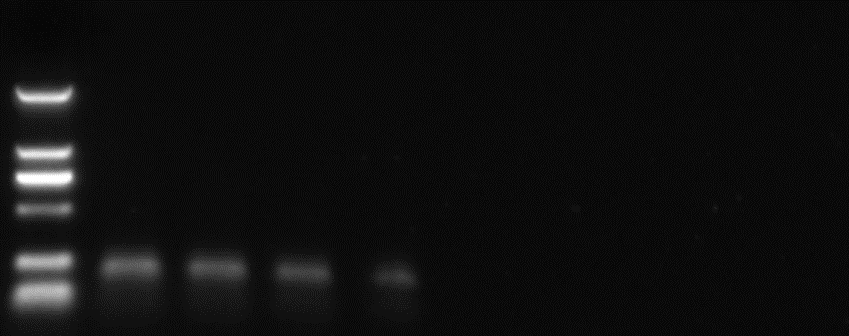


PTPL1 methylation in SU-DHL-4 cells treated with different doses of arsenic disulfide detected by MSPCR.
